# Supplementary material for: Tactile Mechanisms and Afferents Underlying the Rat Pup Transport Response
Source: bioRxiv. 2024 Aug 23:2024.08.23.609194. Preprint. [Version 1] doi: 10.1101/2024.08.23.609194 (PMC11370612; doi:10.1101/2024.08.23.609194)
Supplement: Supplement 1 [file NIHPP2024.08.23.609194v1-supplement-1.pdf]

bioRxiv preprint doi: https://doi.org/10.1101/2024.08.23.609194; this version posted August 23, 2024. The copyright holder for this preprint (which was not certified by peer review) is the author/funder, who has granted bioRxiv a license to display the preprint in perpetuity. It is made available under aCC-BY 4.0 International license.

Supplementary information

Movie 1: Anterior vs Posterior Holding position.
https://figshare.com/s/809990246af6a3c6ab45
25-day old rat pups are shown being held in the anterior position, with grip placed between the ears, for 12 seconds, the pups remain fully immobile. In contrast, when held in the posterior position, the rat pups demonstrated mobility.

Movie 2: 4Hz shaking induces the transport response
https://figshare.com/s/87bd2f87080aa4f28234
25-day old rat pups were subjected to shaking at a frequency of 4 Hz for a duration of 12 seconds to simulate the maternal transport response. The pups were held in the anterior position and demonstrated sustained immobility throughout the stimulation period.

Movie 3: Dual but not single forepaw stimulation mobilizes the pup
https://figshare.com/s/adb055f78e0670019789
25-day old rat pup is shaken at 4Hz for 12 seconds. Stimulation of a single forepaw does not disrupt the pup's immobility. However, simultaneous stimulation of both forepaws results in the pup becoming mobile.

bioRxiv preprint doi: https://doi.org/10.1101/2024.08.23.609194; this version posted August 23, 2024. The copyright holder for this preprint (which was not certified by peer review) is the author/funder, who has granted bioRxiv a license to display the preprint in perpetuity. It is made available under aCC-BY 4.0 International license.

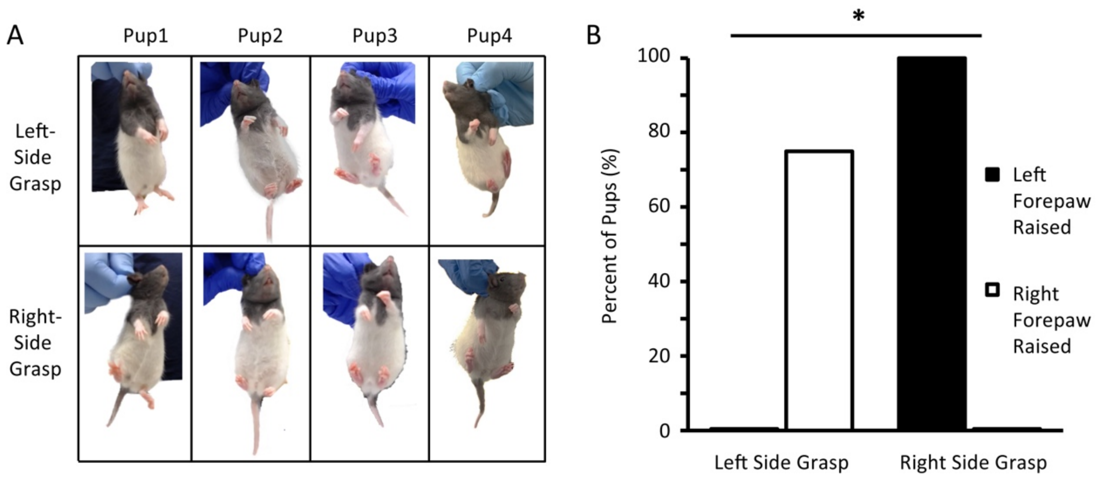

Figure S1 Laterally asymmetric grasping evokes systematic postural adjustments. A. Upper: holding only the left-side of the scruff results in raising of the right forelimb and lowering of the left forelimb. Lower, the reverse is seen when the right-side scruff of the same animal is held (N=4 rats). **B.** Asymmetric arm-raising behavior of pups. Percent is calculated as the number of pups exhibiting the behavior of to the total number of animals. * $p < 0.05$. Statistical analysis was performed using Fisher exact test.

bioRxiv preprint doi: https://doi.org/10.1101/2024.08.23.609194; this version posted August 23, 2024. The copyright holder for this preprint (which was not certified by peer review) is the author/funder, who has granted bioRxiv a license to display the preprint in perpetuity. It is made available under aCC-BY 4.0 International license.

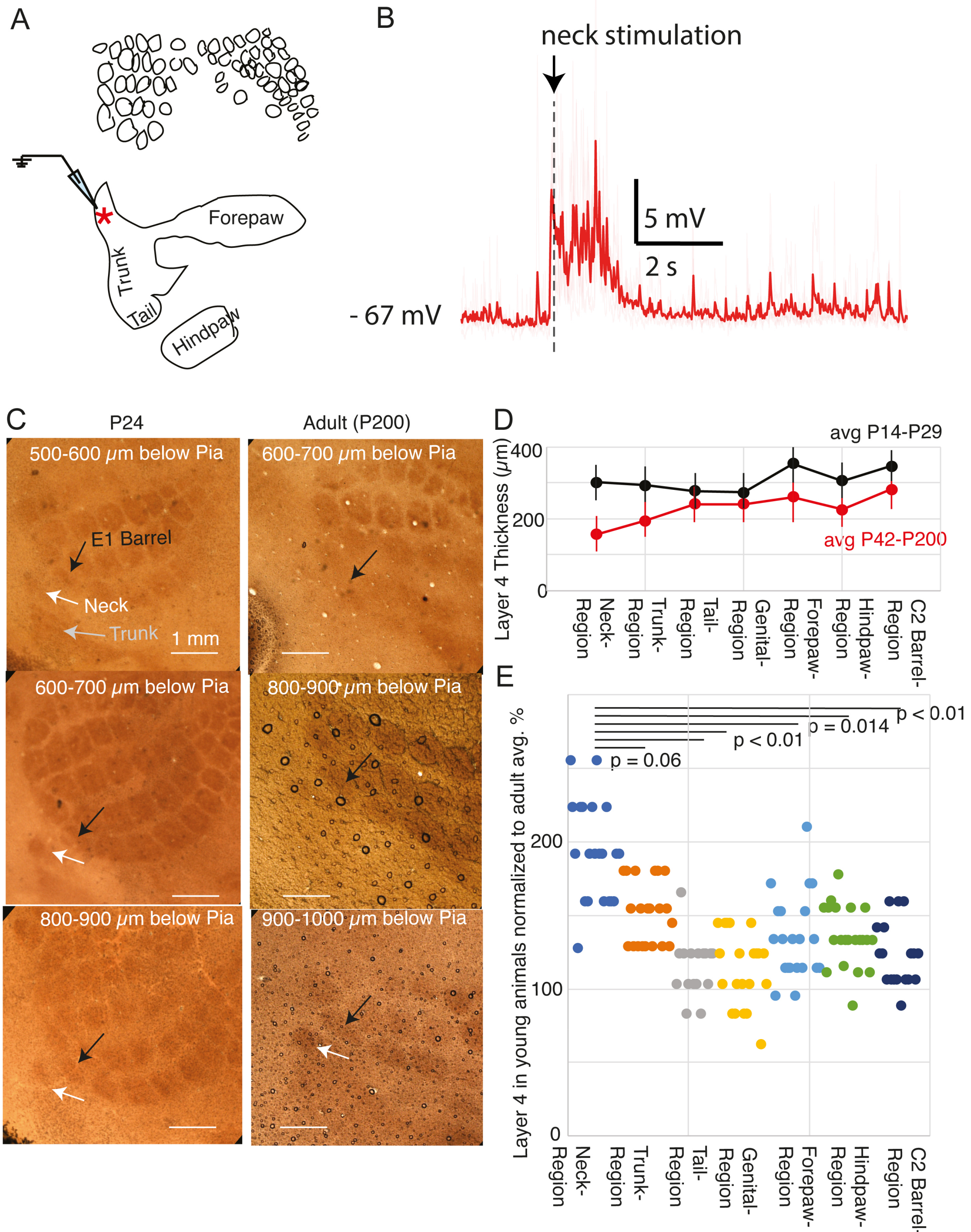

Figure S2 Responses of in rat somatosensory neck cortex and a thinning of somatosensory neck cortex from young to old rats.
A. Line drawing of layer 4 body part contours from a cytochrome oxidase reactivity-stained tangential section through somatosensory cortex of a P24 rat pup, in which we obtained a whole-cell recording of a neuron at the site of the red star; the site was marked by an electrolytic lesion. **B**. Averaged (x5) voltage response to air puffs applied to the neck region from one representative cell in neck somatosensory cortex for a P23 rat pup. The stimulus is a sequence of air puffs at a rate of 4 Hz and comes on at second 2 and continues for 15 s (longer than shown). The responses are clear and habituate. **C**. Loss of cytochrome oxidase reactivity in neck somatosensory from young to old animals. Neck somatosensory cortex layer 4 (white arrow, identified as a dark contour

bioRxiv preprint doi: https://doi.org/10.1101/2024.08.23.609194; this version posted August 23, 2024. The copyright holder for this preprint (which was not certified by peer review) is the author/funder, who has granted bioRxiv a license to display the preprint in perpetuity. It is made available under aCC-BY 4.0 International license.

in the cytochrome oxidase reactivity-stained tangential section through somatosensory cortex) can be identified through three 100 µm sections in young animals (left column). The same holds for the E1-barrel (black arrow) in young animals (left column). In adult animals (right column), however, neck somatosensory cortex layer 4 (white arrow, bottom) can be identified only in one 100 µm section. This differs from the E1-barrel (black arrow) in adult animals (right column), which can be identified in three sections. Scale bars are 1 mm. **D**. Average layer 4 thickness (identified by cytochrome oxidase reactivity) in different regions of somatosensory cortex; error bars refer to standard deviation (SD) in pups (black) and adults (red). **E**. Layer 4 thickness (identified by cytochrome oxidase reactivity) in individual somatosensory cortex maps normalized by average adult layer 4 thickness in the respective region. Neck somatosensory cortex stands out in relative thickness to adults.
